# Phase Change Materials for Energy Efficiency in Buildings and Their Use in Mortars

**DOI:** 10.3390/ma12081260

**Published:** 2019-04-17

**Authors:** Mariaenrica Frigione, Mariateresa Lettieri, Antonella Sarcinella

**Affiliations:** 1 Innovation Engineering Department, University of Salento, Prov.le Lecce-Monteroni, 73100 Lecce, Italy; antonella.sarcinella@unisalento.it; 2 Institute of Archaeological Heritage—Monuments and Sites, CNR–IBAM, Prov.le Lecce-Monteroni, 73100 Lecce, Italy; mariateresa.lettieri@cnr.it

**Keywords:** thermal energy storage (TES), phase change material (PCM), building materials, passive building systems, mortar, concrete

## Abstract

The construction industry is responsible for consuming large amounts of energy. The development of new materials with the purpose of increasing the thermal efficiency of buildings is, therefore, becoming, imperative. Thus, during the last decades, integration of Phase Change Materials (PCMs) into buildings has gained interest. Such materials can reduce the temperature variations, leading to an improvement in human comfort and decreasing at the same time the energy consumption of buildings, due to their capability to absorb and release energy from/in the environment. In the present paper, recent experimental studies dealing with mortars or concrete-containing PCMs, used as passive building systems, have been examined. This review is mainly aimed at providing information on the currently investigated materials and the employed methodologies for their manufacture, as well as at summarizing the results achieved so far on this subject.

## 1. Introduction

The scientific community is severely concerned about the increase of world energy consumption. Global demand for energy is growing rapidly and higher consumption of fossil fuels leads to greater greenhouse gas emissions, particularly carbon dioxide (CO_2_), which contribute to creating heavy environmental impacts, such as ozone layer depletion, global warming and climate change [1].

**Figure 1:**
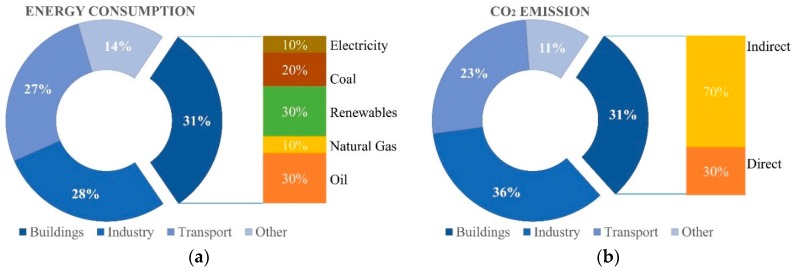
Energy consumptions in the building sector. (**a**) Energy consumption in each sector; (**b**) consequent CO_2_ emissions.

Among all the activities employing a great amount of energy, one of the main sectors in some countries is related to buildings. According to the International Energy Agency (IEA), the building sector accounts for more than 30% of total energy consumption (Figure 1a) and produces around 30% of total CO_2_ emissions (even though when indirect building emissions from power generation are included, buildings and constructions represent nearly 40% of energy-related CO_2_ emissions) (Figure 1b) [2].

The main cause for the intensified energy consumption is the overall change in the living standards and comfort demands for heating in cold regions and cooling in hot ones [3]. As a consequence, the energy efficiency of buildings is today a primary objective of policies regarding energy at regional, national and international levels [4]. The development of novel building materials able to improve the efficiency in energy utilization in the buildings is gaining increasing interest in industrial and academic communities. In addition, thermal energy storage (TES) is a useful tool for improving energy efficiency and increasing energy savings. 

There are three ways to store thermal energy: chemical heat (CH, by breaking and forming molecular bonds), sensible heat (SH, by heating and cooling a material) and latent heat thermal energy storage (LHTES, by melting and solidifying a material) [5]. According to the literature, LHTES is the most attractive approach, due to its high storage capability and small temperature variations from storage to retrieval. In such a system, energy is stored during melting and recovered during freezing of a Phase Change Material (PCM) [6]. 

**Figure 2:**
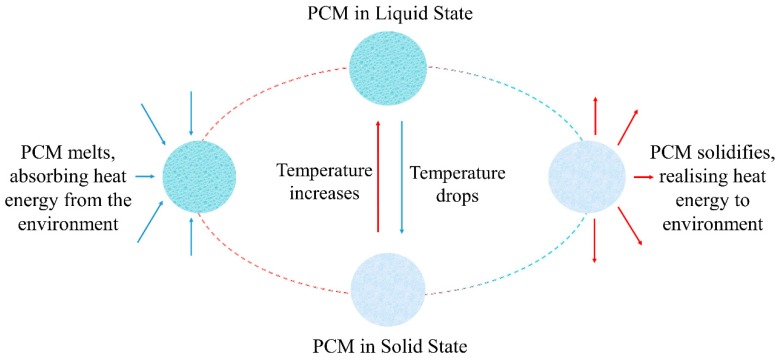
Phase change processes taking place in phase change materials (PCMs).

Due to its capability to absorb and release energy from/in the environment, a PCM has the ability to reduce temperature variations. The operating principle of PCMs takes advantage of the modification of their state due to changes in temperature: as the temperature increases, the PCM passes from the solid to the liquid state, thus, absorbing and storing energy. Conversely, when the temperature decreases, the material can release the previously stored energy, passing from the liquid to the solid state, as illustrated in Figure 2 [7].

The incorporation in building materials of a suitable PCM can reduce the temperature fluctuations, thus, leading to an improvement in human comfort and a reduction in the consumption of energy in the building [8,9]. The use of PCMs in building materials is beneficial, especially in extremely hot and cold climates, where the energy required to maintain the internal conditions of buildings at a comfortable level can achieve significant consumption levels [10].

One of the oldest research on PCMs, describing the application of these materials in buildings, was published by Telkes [11] in 1975, followed by another work authored by Lane [12] in 1986. During the last decades, the integration of PCMs in building materials has gained renewed interest.

The PCMs in buildings are used in walls, floors, and ceilings, or in other building components (e.g., shutters and windows) as well as in heat and cold storage units [4,13]. Most of the applications in building structures consist of wallboards containing a PCM or in the incorporation of a PCM in a concrete or mortar matrix. The study of mortars developed using different binders with the incorporation of PCM largely interested the scientific community. The resulting properties of these new materials have been widely investigated, with a special focus on the thermal behavior in order to assess the advantages in terms of thermal energy storage [14].

This paper presents a literature review of the recent research works dealing with PCMs, taking as an example the production of mortars or concrete-containing phase change materials. After a general summary of the classification and properties of PCMs, the incorporation methods and the applications in the building sector are illustrated. The characteristics and features of different PCMs in mortar or concrete are, then, introduced, highlighting the main differences between the various mortars containing PCMs and describing the different methods employed for their production.

## 2. Classification of PCMs

**Figure 3:**
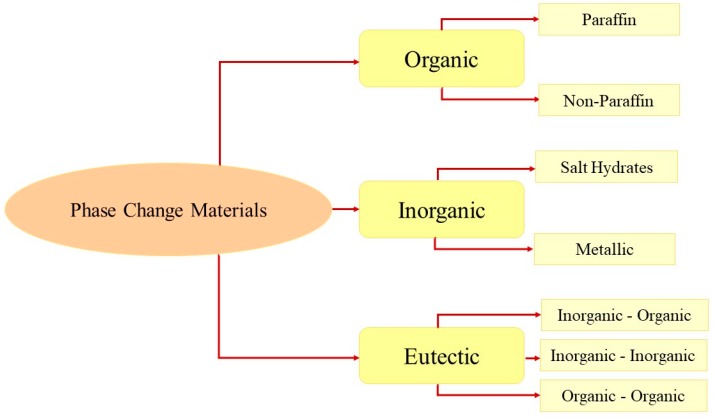
Classification of PCMs.

The first classification of materials used for thermal storage appeared in 1983 and was proposed by Abhat [15]. Based on chemical composition, a PCM can be classified as an organic, inorganic or eutectic compound (Figure 3).

Organic materials, in turn, can be paraffinic or non-paraffinic. Typically, they can change their state several times without displaying any degradation. Salt hydrates and metals belong to a class of inorganic materials. The eutectic mixtures result from the combination of two or more organic and/or inorganic compounds, with the transition temperatures that can meet specific demands [16,17]. In Table 1, the main features of PCMs are summarized. The advantages and disadvantages of each class of PCMs are described in Table 2 [18,19].

It must be underlined, however, that not all existing PCMs can be used for thermal storage in building applications [20]. In order to be suitable for this use, at least two important requirements must be fulfilled: the PCM must display (i) appropriate melting temperature and (ii) melting heat.

The melting heat is a measure of the thermal energy that a material absorbs when it changes its state from solid to liquid. The thermal storage capacity of a PCM is strictly correlated to its melting heat [21].

Referring to the melting temperature, materials with a melting/freezing temperature between 18 °C and 40 °C are particularly suitable as PCM for building applications; this range of temperatures is, in fact, considered to be an optimum. According to the literature, the temperature of phase transition of the selected PCM should be very close to the human comfort temperatures (i.e., 22–26 °C) [22,23]. Nevertheless, PCMs that fall within three temperature ranges have been suggested for use in buildings [16]:

▪ Up to 21 °C for cooling applications;
▪ From 22 °C to 28 °C for optimal human comfort;
▪ From 29 °C to 60 °C for hot water applications, such as in the case of radiant floors often using water combined with PCMs.

The PCMs more suitable for building applications are summarized in Table 3, with the indication of the melting temperature and heat [8,13,14,19,24,25,26,27,28,29,30,31,32,33,34]. Only the materials with phase change temperatures ranging from 18 °C to 40 °C have been reported.

## 3. Properties of PCMs

Among all possible candidates, the most appropriate PCM for a specific application must be selected taking into account some characteristics that will determine its effectiveness. For any thermal energy storage application in buildings, in fact, a careful examination of the overall properties of a PCM should be made, comparing the advantages and disadvantages displayed by each available system and, possibly, admitting a certain degree of compromise.

**Figure 4:**
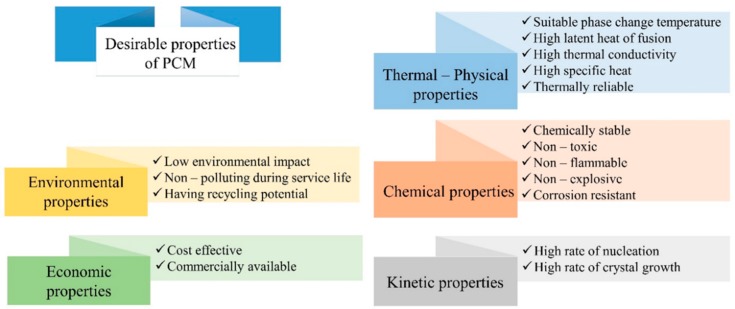
Advantageous properties of PCMs employed in building applications.

The main attractive properties and characteristics that a PCM shoud possess are reported in Figure 4 [16,19].

## 4. PCMs in Building Materials

Although this review mainly focuses on passive building systems for thermal energy storage based on the integration of PCM in building materials, a short overview of all the available solutions is presented.

Generally speaking, the possible introduction of PCMs in building materials is described as follows [4,5,34,35,36].

- Free cooling. This system requires a storage unit to accumulate the thermal energy and use it in heat absorption and in heat release. In this way, the storage medium is used to maintain a cold temperature, when the ambient temperature is lower than room temperature. This process is carried out during the night; the cold air flows through the storage unit, removes heat from the liquid PCM through an electrical fan; at this point, the PCM starts to solidify. When the room temperature rises above a comfortable level, the cold stored in PCM is released. Thus, the PCM absorbs heat from the air, starting the transformation from solid to liquid state [23,26,30,37,38].
- Peak load shifting. This method is based on the use of PCMs that shift the peak energy request far from the peak hours of electrical demand; the peak load may be split throughout the day reducing the highest peaks [5,13,39]. The cooling/heating stored in off-peak hours is used during an on-peak load [40]. Peak cooling load reductions can range from 10 to 57% [4].
- Active building systems. The storage capability of PCMs can be used in systems such as solar heat pump systems, heat recovery systems, and floor heating systems. An example of incorporating PCMs in an active system is radiant floors [5]. These systems consist of a lightweight piped radiant floor, where an integrated PCM layer is aimed at buffering internal gains during the summer season without affecting the winter warming capacity [22].
- Passive building systems. For passive applications, PCMs are integrated into building materials to increase their thermal mass. The incorporated PCM melts during the daytime and solidifies during the night: this process can warm the environment during the day.

### 4.1. Typical Applications

Among all potential applications of PCMs in buildings, the incorporation in construction materials (passive building system), aimed at modifying their thermal properties, has proven to be the most interesting. The combination of building materials with PCMs is an efficient way to increase the thermal energy storage capacity of construction elements. Thereby, wallboards, floors, roof, concrete and other parts are integrated with PCMs in order to improve the thermal performance of the building. The most common solution for implementing PCMs in buildings is the installation of PCM into the interior side of the building envelope. Thus, the use of suitable PCMs in the interiors of the construction allows to absorb and release heat in any room during a large part of the day. Several experimental investigations showed how this strategy positively affects indoor climate and energy use.

Wallboards or plasterboards are very suitable components for the incorporation of PCMs [3,4,5,19,34,41,42]. These elements are cheap and widely used in building applications, especially in lightweight constructions, to reduce the internal air temperature fluctuations. The PCMs can be incorporated in the panel in different ways, as described below.

A PCM can also be added to conventional and alveolar bricks in constructions [3,42].

To insert a PCM in a house roof, a sequence of panels is usually used [3,4,5,13,34,42], each one containing the PCM, or a layer of mortar/concrete, with frustum holes filled with the PCM [3,4,42,43]. The PCM placed in the roof can absorb both the incoming solar energy and the thermal energy from the surroundings; hence, it reduces the internal temperature fluctuations.

The floor is another part of the building that offers a large surface and, thus, a great storage capability. As already described, the floor can often act as an active building system, but it can also be employed in passive ones. In some applications, in fact, PCMs have been included in the concrete layer placed under the floor; PCM panels have been also employed as an overlay to substitute the floor [4,5,13,39,41,42,44]. Advantageous effects are obtained by integrating PCMs in a floor, since a great amount of energy is usually lost from the floor, due to the heat transfer with the ground.

Emerging solutions are those in which PCMs are placed in windows and sunshades [3,4,5,45,46]. In such applications, a PCM must fill the glass, frame, layer, or any other hollow part, such as the cavity of the shutters. The main issue of this application is due to the lack of transparency of PCMs in both their liquid and solid states. Hence, the windows using such systems are blurry, with a reduced transmission of daylight and solar radiation.

Finally, PCMs in mortars or plasters are also considered for interior finishing on walls and ceilings in residential buildings [3,4,5,19,41,42,47].

### 4.2. Methods of Incorporation

Using one or more of the described elements in a house, a significant improvement in energy efficiency is achieved. Such an approach in construction allows to activate thermal inertia and heat storage capability of each room, reducing the internal temperature fluctuations and improving the level of indoor thermal comfort [48].

Different methods have been used to incorporate PCMs in building materials, such as:

▪ Direct incorporation;
▪ Immersion;
▪ Use of micro or macro encapsulated PCMs;
▪ Addition of shape-stabilized PCMs;
▪ Addition of form-stable PCM composites.

It must be emphasized that the terms shape-stabilized and form-stable PCMs have been often considered as synonymous; the two methods, on the other hand, have some distinct characteristics, as following described [19].

The first research published on PCMs largely focused on their direct incorporation and immersion. Direct incorporation is the simplest, practical and economical method: in this case, in fact, the PCM is directly mixed with the construction material [4,45]. The PCMs, in liquid or powdered form, are added to a mixture of materials (such as lime, gypsum, cement paste or concrete) during its production. The main advantage lies in the simplicity and inexpensiveness of the procedure, since no extra equipment is required. However, some problems due to the leakage of PCM, when it is in its melting state, can occur, possibly leading to low fire resistance of the impregnated materials and even causing incompatibility between the mixed materials [3,17]. 

Referring to the immersion method, porous construction materials are immersed in the melted PCM; thus, the porous materials absorb the product by capillary rise [22]. Once again, the mechanical and durability properties of the construction elements can be affected by leakage of PCMs and its incompatibility with the substrate. In particular, the leaked PCM in contact with the cementitious binder may interfere with the hydration reactions [19,27,44]; it may also cause the corrosion of reinforcing steel that, in turn, affects the service life of the concrete structure [49].

Some organic PCMs are not stable under an alkaline environment, which is typical of concrete, and they can easily react with calcium hydroxide; this can lead to a modification of the PCM, with a consequent decline in its properties. Furthermore, if a reaction with PCM occurs during the curing process of fresh concrete, the hydration of the cement may be delayed or interrupted, with a consequent reduction in its final strength. To avoid such unwanted effects, the Portland CEM I was identified as the most appropriate cement to be used with a PCM [50]. 

In order to reduce any interference with the building materials, a well-established approach is the encapsulation of PCMs in a suitable shell material [51]. 

**Figure 5:**
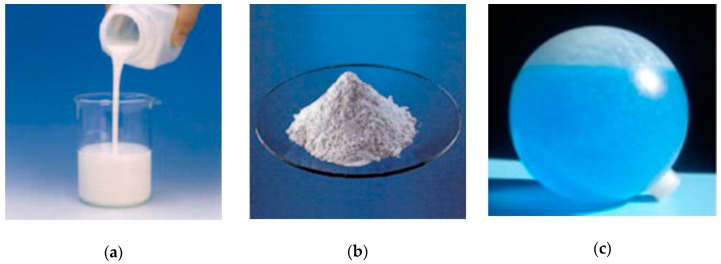
Examples of commercial PCMs: (**a**) micro-encapsulated PCM dispersed in a liquid; (**b**) micro-encapsulated PCM in powder form; (**c**) macro-encapsulated PCM in spherical form. Reprinted with permission from [5]. Elsevier (2015).

Two encapsulation approaches are reported in literature for PCMs: micro-encapsulation (Figure 5a,b) and macro-encapsulation (Figure 5c) methods [16,52]. In order to guarantee a long-term stability of the whole system, the PCM and the container material should display no chemical interactions, irrespective of the encapsulation method [53].

In the micro-encapsulation method, small PCM particles, ranging from 0.1 μm to 1 mm, are wrapped in a thin solid shell. The latter is usually constituted by natural or synthetic polymers [54]; in general, a shell of a high molecular-weight polymer is used. The employed polymer must be compatible with both the PCM and the construction material in which it is applied [50]. Different physical and chemical methods have been developed for the production of micro-encapsulated PCMs [3,35]. The physical methods include: pan coating, air-suspension coating, centrifugal extrusion, vibrational nozzle and spray drying. The chemical methods are: coacervation, complex coacervation and interfacial methods [13,14,27,32,33,52,53,55,56,57,58,59]. Chemical methods are likely to produce much smaller encapsulated PCM particles in comparison with the micro-encapsulated PCMs produced by physical methods [25].

The micro-encapsulation techniques present several advantages over the other procedures previously described [3,16]. These methods allow a reduction in leakage of PCM during its phase transition; they provide a high rate of heat transfer due to a large surface area per unit volume; they can improve chemical stability and thermal reliability (the latter representing the capability to repeat many times the melt/freeze cycle without the occurrence of degradation phenomena) [51]. Furthermore, the previous listed aspects contribute to expand the possibilities of integration of PCMs in construction materials [60]. Nonetheless, some issues still exist: the rigidity of the shell prevents natural convection, thus, reducing the rate of heat transfer [22,28]; micro-encapsulation may affect the mechanical properties of the building material [45]; finally, the very high costs limit this technique to high-value applications [61].

In the macro-encapsulation method, a significant amount of PCM is stored in rigid containers, such as tubes, spheres or panels, that are subsequently introduced in the construction elements. It is possible to produce, therefore, a system easier to move and handle and properly designed to satisfy the required application. The PCMs obtained with macro-encapsulation usually have poor thermal conductivity and the tendency to solidify at the edges. These materials need protection (for instance against drilling holes in the walls) and require that their introduction in the structure is performed in situ [3,19,60].

Among the different PCM encapsulating methods, the shape-stabilization one proved to be a very promising technique, although very complex in the implementation [35]. The shape-stabilized PCMs display several advantages, such as: large apparent specific heat, suitable thermal conductivity, the ability to maintain the shape during the phase-change process; moreover, they are thermally reliable over a longer service period, thus, the melt/freeze cycle responsible for the phase change behavior can be efficiently repeated many times. Amounts of PCM up to 80%, as a percentage of the total weight, can be used with this technique [3,13,17,62,63]. 

The shape-stabilized PCMs can be obtained by physical methods (such as blending, adsorption, and impregnation) or chemical methods (including graft copolymerization and sol-gel methods) [33]. The material supports used to fabricate shape-stabilized PCMs may consist of organic materials, based on polymers, such as high-density polyethylene (HDPE), styrene and butadiene, but also of inorganic porous materials [55]. The supports are usually able to prevent the leakage of the PCM [27]. The use of waste materials or by-products of different industrial processes may improve the sustainability of this technique [64,65].

**Figure 6:**
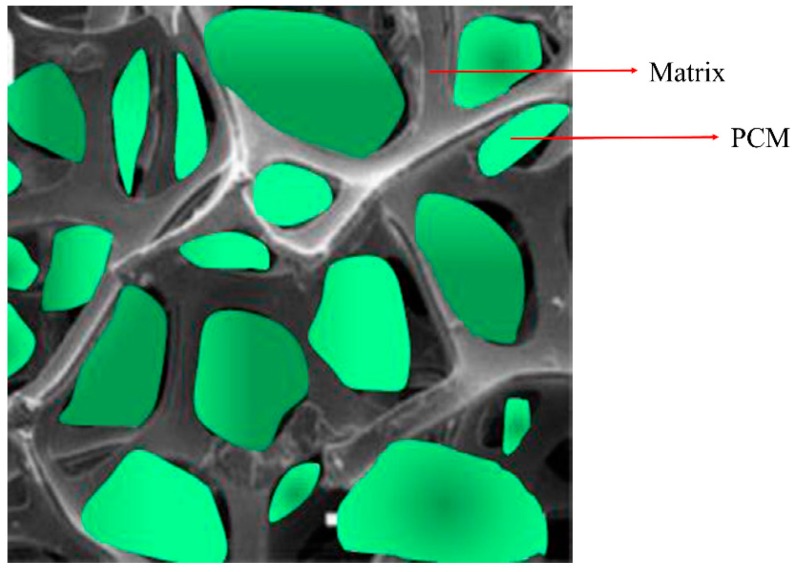
Example of shape-stabilized composite PCMs. Adapted with permission from [55]. Copyright 2015 Elsevier.

In the case of polymers, the PCM and the supporting material are melted and mixed at high temperatures. Then, the polymeric support is cooled below its glass transition/melting temperature, solidifying, while the PCM, that is still in the liquid state, fills the empty space of the support (Figure 6).

In order to improve the thermal conductivity of shape-stabilized PCMs, some additives can be added [19]. In particular, the shape-stabilized PCM composites show enhanced thermal conductivity with the incorporation of carbon-based nanostructures (CNs), namely: expanded graphite (EG), compressed expanded natural graphite (CENG), nano-graphite (NG), exfoliated graphite nanoplatelets (xGNPs), graphene, nitrogen-doped graphene (NDG), graphene oxide (GO) or multi-wall carbon nanotubes (MWCNTs).

**Figure 7:**
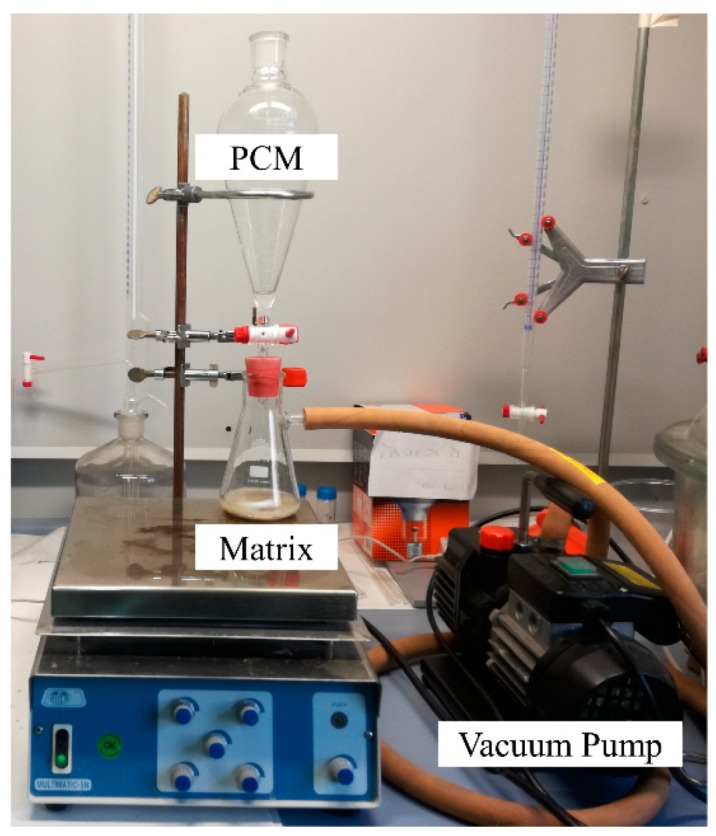
Vacuum impregnation equipment used to prepare form-stable PCM composites.

A porous matrix of inorganic (such as silica-based material, perlite, diatomite, silica dioxide, clay materials) can act as the support containing the form-stabilized PCM [18,62,66,67,68]. In this case, the form-stable PCM composite can be obtained by immersing the matrix in the liquid PCM; a vacuum system can be employed to force the impregnation. Various vacuum impregnation systems have been used to prepare form-stable PCM composites [19]; an example is shown in Figure 7.

The term form-stable composite PCM is specifically used to define a composite material retaining an optimum/maximum percentage of PCM and showing no leakage when the temperature of the composite approaches the melting point of the PCM [69]. This feature also extends the durability of the material, preventing its degradation upon thermal cycling. This represents an important advantage for applications requiring long-term performance, like those related to buildings [63].

In Table 4, the advantages and disadvantages of each incorporation method are briefly described.

## 5. PCMs in Mortars: Potential and Issues

The incorporation of phase-change materials in mortars employed in the interiors of buildings appears the most attractive solution in an attempt to minimize the massive energetic consumption related to building conditioning. Such an approach allows the regulation of the temperature inside buildings through latent heat energy storage, using only solar energy as a resource, thus, reducing the need of heating/cooling equipment. Incorporation of PCMs in mortar and concrete can be an efficient method due to the large heat exchange area surfaces; furthermore, the final functional material can be adapted in a wide variety of shapes and sizes. Being mortar and concrete widely used as construction materials, such PCM composites can be employed in any practical application. In addition, quality control can be easily achieved in the produced materials.

In recent years, the research on mortars containing PCMs has been mainly focused on cement and gypsum compositions, due to the good mechanical and thermal properties of these binders. At first, cement-based mortars employed in masonry were taken into consideration and PCMs incorporated into these cementitious systems were examined, analyzing their capability to improve the energy efficiency of building envelopes. The results of these studies have shown that the incorporation of PCM appreciably decreased the mechanical properties of the composite, especially in the case of cement pastes [70,71,72,73]. For such a reason, the research has moved towards the investigation on mortars for interior and/or exterior coatings, where high values of mechanical strength are not required. More recently, other PCM-mortar systems based on different binders, such as aerial lime [73,74,75,76,77,78,79,80,81,82,83,84,85,86,87], hydraulic lime [71,88,89], and, in some cases, geopolymers [90,91,92,93], have been developed and studied [74,78,80,83,90,92].

Generally speaking, both the amount of heat stored as well as the thermal conductivity of the final material increased upon the addition of a PCM in a mortar [75,89,90,91,94,95,96]; the PCM reduces the fluctuations in temperature and the use of the composite material can efficiently improve the indoor temperature comfort [79,97,98,99]. The concurrent use of other additives (e.g., TiO_2_ nanoparticles) allows to obtain multifunctional materials [95] to prevent the accumulation of dirt and growth of microorganisms on the surfaces; they can even degrade pollutants.

### 5.1. Effects of PCM’s Type and Content

It has been found that the higher the PCM content, the better the heat storage capacity of the mortar [100,101]. Comparing the heat storage in samples with and without PCM incorporation, the PCM composite can reduce the heat effect in the heat storage process as well as enhance the heat effect in the heat release process.

Generally speaking, the addition of a PCM in a mortar formulation requires a larger amount of water to guarantee the workability of the fresh mixture [72,87,102,103]. On the other hand, PCMs added as spherical particles can assure an appropriate workability of host mortars over time [95], since this shape appreciably reduces the surface friction. 

Among the different types of PCMs, organic compounds have been found to be the most promising candidates for applications in mortars due to their chemical stability, non-corrosive nature, reproducible melting and crystallization behavior, even after repeated thermal cycles [104,105]. Moreover, more than one material can be used in a mixture, to achieve the desired phase transition temperature [99,106].

In the examined literature, paraffin compounds [65,70,73,74,76,77,78,79,80,81,82,83,84,85,86,87,88,89,90,91,95,97,100,102,107,108,109,110,111,112,113,114,115,116,117,118,119,120,121,122,123,124,125,126,127,128] are the most used PCM in mortars. Furthermore, higher hydrocarbons (like octadecane [72,94,101,129,130,131,132], hexadecane [98], or heptadecane [71]), fatty acids or their mixtures [99,106,133,134], and polyethylene glycols [64,96,135] have been extensively examined for these applications. Their phase change temperature usually ranges between 18 °C and 36 °C, and the phase change enthalpy is between 100 and 260 J/g. Some of these compounds have a low thermal conductivity which limits heat transmission, this latter representing a significant drawback in thermal exchange applications. However, the addition of fillers with high thermal conductivity could solve this problem; for instance, expanded graphite is widely used to increase the thermal conductivity of the PCMs [99,100,101,136].

The incorporation of PCM has been mostly performed by their addition in microcapsule form; their composition, when detailed, is generally based on polymethylmethacrylate or melamine–formaldehyde [67,70,71,72,74,76,78,79,80,81,82,83,84,85,86,87,102,117,121]. Their application through the form- or shape-stabilization methods is increasing [64,96,123,126,127,130,132,133], with perlite [90,106,122,124,125,134] and graphite [94,98,99,100,101] mainly employed as supports.

Salt hydrates have been rarely applied in mortars [137]; in some rare case, the PCM has been added by direct incorporation; in addition, it has been found not appropriate in combination with a cement-based binder.

### 5.2. Influence on the Porosity and Mechanical Properties of the Mortar

Mortars have shown better performance using microcapsules containing a PCM, in amounts from 15 to 20 wt.% (by total mass), with an excellent compromise between mechanical strengths, shrinkage, thermal efficiency, and costs [76,78,82,86,91,113,117]. In such cases, a strong reduction of macroporosity, rather than a decrease of total porosity, leads to an improvement in mechanical strength [76]. Usually, a lower total open porosity and a higher number of smaller pores are obtained in a PCM-modified mortar in comparison to the reference material [87,88,89]. The incorporation of PCM, in fact, produces a filling effect that reduces the number of macropores [76,88]. In a few cases, even though the pore size increases upon the addition of a PCM, the compressive strength rises due to the crystallization of minerals (such as aragonite) able to enhance the resistance of the mortar [75]. Leakage of the PCM into the mortar could increase its porosity [110] with a consequent reduction in mechanical properties. Large amounts of PCM (i.e., higher than 20 wt.%) have been found not useful, due to the great amounts of water required in these mortar mixes [79] causing a high porosity [78]. On the other hand, it has been found that a high PCM content does not necessarily imply an increase in the latent heat transfer [127], proving that the internal porosity plays an important role in this process [65,76,82,83]. The presence of nanopores leads to a decrease in the heat transfer capability, even though the PCM content is close to the optimal value.

**Figure 8:**
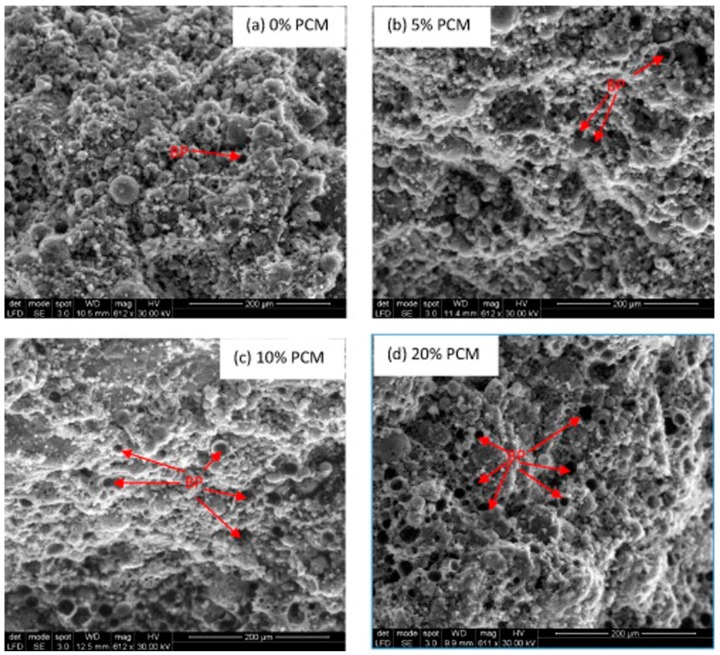
SEM image of failure surface of mortar specimens containing (**a**) 0% PCM, (**b**) 5% PCM, (**c**) 10% PCM, and (**d**) 20% PCM (BP = broken particles). Reprinted with permission from [91]. Elsevier (2015).

The mechanical strength of mortars containing PCMs is strongly related to the microstructural characteristics of the material, that are, in turn, influenced by the nature/dosage of the components and by the amount of added water [74,95]. However, the content of PCM, rather than the type of binder, is the key factor affecting the mechanical behavior of the final mortar. The added PCMs are likely to behave more like voids than as aggregates in mortars and concretes; therefore, they do not contribute to the compressive strength [72]. When mechanical properties lower than those displayed by pristine materials are obtained [73,100,120,127], this result is mainly ascribed to the increased required amount of water due to incorporation of PCM [71,81,95], rather than to the relatively poor interface compatibility [120]. A greater strength is generally obtained when a lower amount of PCM is used [100,112]. A reduction in mechanical properties can also be observed when the addition of PCMs produces a decrease in hydration heat and/or a delay in the hydration kinetics [73,120,126]. Even though the PCM does not take part in the hydration processes, it can sequester water, thus, hindering hydration of the mortar. These effects influence the curing and, as a consequence, the mechanical properties of mortars that, especially at early stages, are lower when a PCM is added [110]. Furthermore, by increasing the content of the PCM, a greater number of incorporated PCM particles can be broken during shearing failure (Figure 8); the so formed void space, previously occupied by the un-broken PCM, causes an increase in the porosity of the matrix [113] and, consequently, lower shear strength and stiffness can be measured [70,91,117].

### 5.3. Main Issues: Shrinkage, Cracking, and Leaking

The addition of microcapsules of PCM significantly affects the shrinkage of the mortar [71,79]; the higher the amount of PCM, the higher the shrinkage. To avoid cracking, the shrinkage phenomena taking place during the curing process must be limited. To this aim, small amounts of PCM and a low water/binder ratio are usually recommended [95]. Some works also examined the capability of PCMs to mitigate early-age temperature rise in cementitious materials caused by exothermic cement hydration and the resultant risk of thermal cracking [138,139,140].

Damage of the PCM particles, taking place even during the initial mechanical mixing, may cause a lower heat capacity [73,119] and can also reduce the resistance of the mortar to fire [110].

The use of lightweight aggregates (LWAs) as a support for the PCM can reduce the interferences with the hydration reactions [123,126] as well as the leakage of PCM from the composite [122]. Furthermore, the addition of fiber and/or gypsum has been found to be a good solution to solve problems related to cracking caused by the incorporation of microcapsules in lime-based mortars [78,79,80,81,82]. The inclusion of stiff quartz can also significantly reduce shrinkage due to the aggregate restraint effects [119].

### 5.4. Analytical Characterization and Simulations by Prototypes

Besides the technical (composition, workability, density, porosity, thermal conductivity) and mechanical characterization, several tests and analytical techniques have been used to evaluate the properties and the behavior of the mortars containing PCMs. In particular, observations through a scanning electron microscope (SEM) have been carried out to control the microstructure of mortars [70,71,76,80,83,113,115,133] and the impregnation of the support [90,130], or to reveal the damage of the encapsulated PCM particles [73,74,110,119,131,132]; this latter event frequently results in the leakage of the PCM.

The thermal characterization is usually carried out by differential scanning calorimetry (DSC). The shape of the DSC curves and the phase change temperatures calculated on the mortars containing PCM capsules and on pure PCM are similar [72,88], with the specific enthalpy proportional to the mass fraction of PCM in the mortar sample [114]. Conversely, the thermal properties of the PCM in a support, measured by DSC, are often found to be lower than those of pure PCM [64,90,96,124,132,134]. The difference observed between temperatures for melting and solidification, indicative of super-cooling, is common in PCMs [120] and it is ascribed to a lack of heterogeneous nucleation sites where the solidification is initiated [119,121]. DSC experiments performed at high heating/cooling rates intensify the differences between the heating and cooling processes, both in terms of the peak temperatures and the shape of the curves [102,114,115,122]. This hysteretic behavior in the thermal response of mortars with PCM is appreciably reduced as the heating/cooling rates are decreased [115].

Thermal analyses are performed to determine the effective content of PCM [94,98,120,130,132] or to evaluate the heat of hydration of the mortar [113]. The influence of PCMs on the hydration reactions and on the amounts of hydration products formed in these processes can be also evaluated by FT-IR (Fourier Transform Infrared Spectroscopy) analysis [70,123]. These latter investigations allow to examine the compatibility and interactions between the PCM and the matrix. Usually, the addition of PCM causes no new signals; only slight shifts of the characteristic absorption peaks of the components are observed. Such a result indicates that no chemical reactions, but only physical interactions, occur and that the integration of the PCM component takes place without a change in the chemical composition of the mortar [64,120].

Mortars containing blends of different micro-encapsulated PCMs, with different melting temperatures, have been investigated [115]. The final properties of such mortars were well predicted through the superposition of the effects of each PCM [114], as long as no interaction occurs between them.

**Figure 9:**
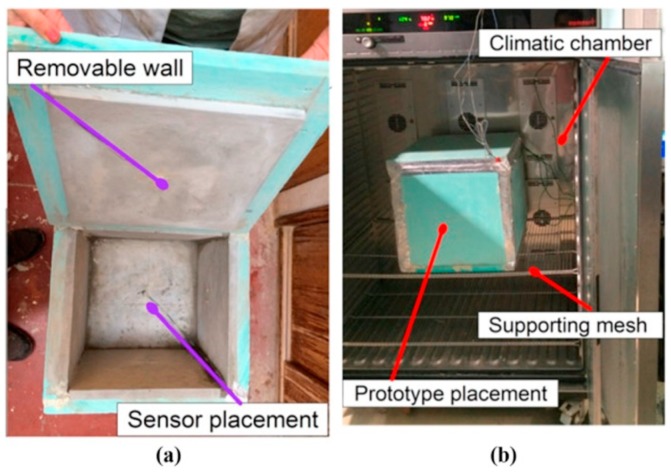
Prototype test cell including a PCM plastering mortar: (**a**) internal walls; (**b**) set-up within the climatic chamber for thermo-hygrometric cycles. Reprinted with permission from [116]. Elsevier (2015).

Prototypes or numerical models have been often proposed to simulate and predict the behavior at the macro-scale level of mortars containing PCM [70,74,107,108,109,111,116,118,121,124,129], mainly in terms of thermo-physical properties of the final system containing PCM and relative cost implications. In most of these studies, the numerical solutions fitted very well with the corresponding experimental measurements, irrespective of the analyzed season. An example of a prototype used to experimentally evaluate the thermal performance of a mortar containing PCM is reported in Figure 9.

### 5.5. Durability

Very recently, different studies have been undertaken to assess the durability of mortars containing PCMs. Several aspects have been investigated; in particular, the structural integrity of the PCM during the production of the mortar composites, the possible interactions between the PCM and the other components, and the matrix durability [99,119]. For this latter evaluation, the resistance to water absorption, freeze/thaw tests, and biological colonization have been mainly used.

Only a few studies have dealt with the durability of PCMs in alkaline cementitious environments [138,141]. During hydration, cement particles dissolve, turning the pore solution into a caustic electrolyte. Indeed, the pore solution contains alkalis species (in particular, ${\mathrm{SO}}_{4}^{2-}$, and ${\mathrm{Ca}}^{2+}$) and, thus, typically exhibits a pH greater than 13 [142]. When micro-encapsulated PCMs are embedded in such caustic systems, chemical reactions between the pore solution and the capsule shell could take place, resulting in modifications and in a reduction in durability. The PCMs seem not to affect the corrosion behavior of reinforced concrete [49]; on the contrary, the incorporation of PCM could enhance the corrosion resistance by forming a protective film on the rebar surface, even if the concrete containing PCM develops a higher porosity compared to the un-modified concrete. The behavior and stability of PCM have been investigated in environments similar to those encountered in mortar and cement through immersion in alkaline solutions (Ca(OH)_2_, NaOH) and in a saturated calcium sulfate solution. These tests highlighted an enthalpy reduction (around 25%) in the materials under study, mainly due to the rupture of the shell capsules with a consequent PCM leakage [119,138].

The low porosity due to the presence of PCM in mortars provides beneficial effects in terms of the composite’s durability, due to the reduction in water absorption and hygroscopic capacity [84,89]. This effect has been ascribed to the ability of the PCM particles to obstruct and interrupt the capillary network [143]. A reduction in porosity can have a positive effect also towards the freeze-thaw resistance [84,122,144]; the incorporation of a PCM, on the other hand, generally results in higher losses of material during temperatures cycles below and above 0 °C.

The presence of additional components can decrease the bulk density, porosity, thermal conductivity and capillary water absorption of mortars, contributing to improve their durability, their resistance to biological colonization and to freeze-thaw action [77,80,85]. In addition, PCMs have been proposed for low-temperature applications as an alternative to de-icing salts, since they can improve the resistance against freeze-thaw cycles [93,145,146,147] and to reduce cracks formation in concrete. PCMs can reduce the time and depth of freezing in concrete by at least 10% [148].

## 6. Economic and Environmental Evaluation of Mortar with PCMs

Some considerations can be done on the overall costs of mortars containing PCM and on their applications in constructions. Starting from the assumption that using PCMs in buildings should lead to a reduction in energy consumption, thus, to an economic saving, there are different aspects to take into account. Usually, inorganic PCMs are cheaper than organic ones; among all the techniques used to incorporate PCMs in building materials, the micro- and macro-encapsulations are costly compared to the other methods (direct incorporation, direct impregnation). The shape- or form-stabilization techniques are promising, but still expensive, unless waste materials are used to produce PCM composite systems.

The incorporation of PCMs in mortars, improving the thermal comfort in the buildings, reduces the need for cooling and heating conditioning, leading to economic advantages. On the basis of numerical or experimental simulations, the energy costs, related to the capability of the mortars containing PCMs to decrease the heating and cooling needs (by increasing the minimum temperatures and decreasing the maximum temperatures), have been estimated to decrease by 10–50% [74,107], depending on the simulated season.

Very few studies report the assessment of applications of mortars with PCM using Life Cycle Analysis (LCA) and/or Life Cycle Cost Analysis (LCCA), used to evaluate the life cycle environmental impact and cost effects, respectively. Even though these techniques are already well developed and standardized, to the best of the authors’ knowledge, there are no national or international standards available to test thermal energy storage products [149,150].

In general, PCMs in buildings can decrease the energy consumption, even though this may not imply an effective reduction in the global environmental impact throughout the lifetime of the structure [151]. Indeed, in most cases, the application of PCMs does not seem economically viable because of the high initial investment cost [149,152,153].

The environmental impact of PCMs could be greater than the conventional construction materials, depending on the type of PCM and the climate [105,154,155]. The benefits of the PCM increase in sites where the weather conditions are similar all year long [156]. Salt hydrates can be compensated in 25 years of use (while alkanes in 61 years) [150]. However, at the end of their useful life, most PCMs can be recycled; organic PCMs are biodegradable, and inorganic ones are innocuous. However, in order to minimize the overall environmental impact, the use of long-lasting PCMs, the extension of the useful life of the building, as well as the development of new PCMs with very low environmental impact, are suggested [156]. The use of PCM in heating and cooling systems in combination with conventional systems can further reduce the environmental impact.

## 7. Outlook for Future Works

Future research works on mortar/concrete containing PCMs are still needed to refine the methods of preparation, mitigate the strength reduction and overcome the durability issues. Possibly, different sustainable, green PCMs/support couples should be identified along with eco-efficient and low costly methods for the production of mortar/concrete containing PCMs. Very few studies have investigated the utility of geopolymers, even though this kind of mortar is eco-friendly and offers economic advantages, since industrial by-products and mine wastes can be used for their production.

Further studies should also be focused on economic and environmental assessments due to the presence of PCMs in building materials, since the current state-of-the-art about these topics is still lacking.

Experimental studies conducted in the field would be useful to document the performance and potential of the PCM in a real context. On the basis of these tests in real full-scale buildings, numerical models to assess the advantages and disadvantages of structures with or without PCMs (in advance) could be elaborated. Moreover, it would be desirable to develop simple but certified simulation tools that easily replicate the service conditions of PCM integrated into mortar or concrete in order to assess, using few tests, both the performance and durability of these materials.

## 8. Conclusions

In this paper, the use of PCMs in building materials was reviewed, especially for passive building systems, with the aim to outline their advantages in terms of thermal effects and thermal efficiency. In the first part of the paper, the main characteristics of PCMs were reported, describing the different and possible ways to introduce them in building materials. In the second part, a review of recently published experimental is presented, focusing on examples of PCMs introduced in mortars or concrete. Through this literature survey, the used materials and the supports, the preparation procedures (i.e., the different methods used to incorporate PCMs in building materials), the tests performed to analyze the final product, and the main results obtained in these studies are presented and discussed.

Thus, the following conclusions can be drawn.

▪ As far as the current world energy consumption is concerned, it is important to find alternative ways of saving energy and preserving the environment.
▪ PCMs are generally considered efficient materials that can improve thermal comfort in a building.
▪ The selection of the appropriate PCM for a specific construction material and/or a definite application must start from its properties (thermo-physical, chemical, functional, environmental and economic).
▪ Among all the available methods to incorporate PCMs in building materials, the micro-encapsulation is the most used due to its advantages; however, in the last years, the form-stabilization method has gained popularity thanks to its low costs of production. Moreover, the latter is a promising technique due to the possibility to employ waste materials as a support for PCMs.
▪ Concretes and mortars are considered suitable construction materials to incorporate PCMs since they are largely present in building constructions; furthermore, mortars can be applied in a building even after its construction.
▪ The most widespread PCMs in building materials are organic in nature and have a melting temperature in a range between 20° and 40 °C.
▪ The performance of micro-encapsulated PCM and those of pure PCM have been scarcely compared so far.
▪ Chemical properties, thermal properties, and thermal stability are the main properties analyzed for PCMs. On the other hand, mortars/concretes with the addition of PCMs have been mainly studied in terms of their morphology, mechanical properties and thermal conductivity.

## Figures and Tables

**Table 1:** Main features of the different class of PCMs.

Type of PCM	Composition	Melting Temperature (°C)	Heat of Fusion (J/kg K)	Cost
Organic	Paraffin	−12–71	190–260	Costly
	Non-Paraffin	8–187	130–250	Highly costly
Inorganic	Salt hydrates	11–120	100–200	Low Cost
	Metallic	30–96	25–90	Costly
Eutectic	Paraffin	4–93	100–230	Costly
	Non-Paraffin	−12–71	190–260	Costly

**Table 2:** Main advantages and disadvantages of each type of PCMs.

Type of PCM	Advantages	Disadvantages
Organic	▪ Available in a large temperature range; ▪ Chemically inert; ▪ Do not undergo phase segregation; ▪ Thermally stable for repeated freeze/melt cycles; ▪ Low vapor pressure in the melt form; ▪ Relatively small melting heat; ▪ Non-corrosive, or mildly corrosive (fatty acids); ▪ Compatible with construction materials; ▪ Small volume change during phase transitions; ▪ Little or no super-cooling effect during freezing; ▪ Innocuous (usually non-toxic and non-irritant; non-paraffin type may have various levels of toxicity); ▪ Stable below 500 °C (non-paraffin type shows instability at high temperatures; ▪ Recyclable.	▪ Low thermal conductivity (around 0.2 W/m K); ▪ Moderately flammable; ▪ Non-compatible with plastic containers.
Inorganic	▪ High volumetric storage heat; ▪ High melting heat; ▪ High thermal conductivity (0.5 W/m K); ▪ Cheap and readily available; ▪ Nonflammable; ▪ Compatible with plastic containers; ▪ Sharp phase change; ▪ Low environmental impact; ▪ Potentially recyclable.	▪ Super-cooling during freezing; ▪ Phase segregation during transitions; ▪ Corrosive to metals; ▪ Irritant; ▪ High vapor pressure (inducing water loss and progressive changes in thermal behavior during thermal cycles); ▪ Low durability (possible long term degradation when exposed to environmental agents); ▪ Moderate chemical stability; ▪ High volume change.
Eutectic	▪ Sharp melting temperature; ▪ High volumetric thermal storage capability (slightly lower than organic PCMs).	▪ Limited data available on their thermo-physical properties.

**Table 3:** PCMs for building applications: composition, category, melting temperature and melting heat.

PCM	Type	Melting Temperature (°C)	Melting Heat (J/kg K)
Glycerin	O	18	198.7
Hexadecane	O	18.1	236
KF∙4H_2_O	I	18.5	231
Butyl stearate	O	19	140
Propyl palmitate	O	19	186
Paraffin C_16_–C_18_	O	20–22	152
Heptadecane	O	20.8–21.7	171–172
Dimethyl sebacate	O	21	120–135
Octadecyl 3-mencaptopropylate	O	21	143
Lithium chloride ethanolate	O	21	188
FeBr_3_∙6H_2_O	I	21	105
Paraffin C_17_	O	21.7	213
Erythritol palmitate	O	21.9	201
Polyglycol E600	O	22	127.2
Isopropyl stearate	O	22.1	113
Paraffin C_13_–C_24_	O	22–24	189
34%C_14_H_28_O_2_ + 66%C_10_H_20_O_2_	E	24	147.7
50%CaCl_2_ + 50%MgCl_2_∙6H_2_O	E	25	95
Octadecane + docosane	E	25.5–27	203.8
Mn(NO_3_)_2_∙6H_2_O	I	25.8	125.9
Octadecane + heneicosane	E	25.8-26	173.93
Octadecyl thioglycolate	O	26	90
Lactic acid	O	26	184
1-Dodecanol	O	26	200
50%CH_3_CONH_2_+50%NH_2_CONH_2_	E	27	163
Vinyl stearate	O	27–29	122
Paraffin C_18_	O	28	244
Octadecane	O	28–28.1	244–250.7
Methyl palmitate	O	29	205
CaCl_2_∙12H_2_O	I	29.8	174
CaCl_2_∙6H_2_O	I	29-30	171–192
LiNO_3_∙3H_2_O	I	30	296
Ga	I	30	80.9
47%Ca(NO_3_)_2_∙4H_2_O + 53%Mg(NO_3_)_2_∙6H_2_O	E	30	136
Capric acid	O	30.1	158
60%Na(CH_3_COO)∙H_2_O + 40%CO(NH_2_)_2_	E	30–31.5	200.5–226
Tridecanol	O	31.6	223
Na_2_SO_4_∙10H_2_O	I	31–32.4	251.1–254
Na_2_SO_4_∙3H_2_O	I	32	251
Na_2_CO_3_∙10H_2_O	I	32–36	246.5–247
CaBr_2_∙6H_2_O	I	34	115.5
LiBr_2_∙2H_2_O	I	34	124
Zn(NO_3_)_2_∙6H_2_O	I	35–36	265–281
Na_2_HPO_4_∙12H_2_O	I	36–36.4	146.9–147
FeCl_3_∙6H_2_O	I	37	223
Tetradecanol	O	37.8	225
Camphenilone	O	39	205
Docasyl bromide	O	40	201
Caprylone	O	40	259

O = Organic; I = Inorganic; E = Eutectic.

**Table 4:** Advantages and disadvantages of different methods for incorporation of PCMs into building materials.

Method of Incorporation	Advantages	Disadvantages
Direct incorporation	Simple and cheap	Possible leakage of PCM in the melting state; flammability of the impregnated elements is possible, as well as incompatibility between the materials.
Direct impregnation	Simple, practical and cheap	Leakage and incompatibility can occur, affecting the mechanical properties and durability of the construction elements.
Micro-encapsulated PCM	Reduced leakage of PCM during phase transition; higher heat transfer rate; improved chemical stability and thermal reliability.	The capsules are expensive; their rigidity may prevent natural convection and reduce the heat transfer rate; the mechanical properties of the construction materials may be affected.
Macro-encapsulated PCM	A significant quantity of PCM is packed in the container; easiness and suitability for any specific application.	Poor thermal conductivity and tendency to solidification at the edges; introduction in the structure must be carried out in situ.
Shape-stabilized PCM	Large apparent specific heat; suitable thermal conductivity; ability to maintain the shape of PCM during the phase-change; thermal reliability over a long period of time; reduced leakage phenomena.	Complex equipment is needed for their preparation; need to use additives to improve the thermal conductivity.
Form-stable composite	Very cheap; retaining of high amount of PCM without leakage above its melting point.	Complex equipment is needed for their preparation.
